# Differential Chromosome Conformations as Hallmarks of Cellular Identity Revealed by Mathematical Polymer Modeling

**DOI:** 10.1371/journal.pcbi.1004306

**Published:** 2015-06-01

**Authors:** Imen Lassadi, Alain Kamgoué, Isabelle Goiffon, Nicolas Tanguy-le-Gac, Kerstin Bystricky

**Affiliations:** 1 University of Toulouse, UPS, Toulouse, France; 2 Laboratoire de Biologie Moléculaire Eucaryote, CNRS, LBME, Toulouse, France; Rutgers University, UNITED STATES

## Abstract

Inherently dynamic, chromosomes adopt many different conformations in response to DNA metabolism. Models of chromosome organization in the yeast nucleus obtained from genome-wide chromosome conformation data or biophysical simulations provide important insights into the average behavior but fail to reveal features from dynamic or transient events that are only visible in a fraction of cells at any given moment. We developed a method to determine chromosome conformation from relative positions of three fluorescently tagged DNA in living cells imaged in 3D. Cell type specific chromosome folding properties could be assigned based on positional combinations between three loci on yeast chromosome 3. We determined that the shorter left arm of chromosome 3 is extended in *MAT*α cells, but can be crumpled in *MAT* **a** cells. Furthermore, we implemented a new mathematical model that provides for the first time an estimate of the relative physical constraint of three linked loci related to cellular identity. Variations in this estimate allowed us to predict functional consequences from chromatin structural alterations in *asf1* and recombination enhancer deletion mutant cells. The computational method is applicable to identify and characterize dynamic chromosome conformations in any cell type.

## Author Summary

The spatial organization of the genome inside eukaryotic cell nuclei has been shown to play a role in transcription, replication, recombination and DNA repair. Probabilistic models have correlated structural fluctuations with these processes, but methods to detect transient features describing chromosome conformation are lacking. We developed a new fluorescent repressor-operator system (FROS) based on the λ repressor and combined it with the existing lac and tet FROS to measure the relative spatial positioning between three labelled DNA loci on chromosome 3 in live yeast cells. To quantitatively analyze and interpret the data we applied an original computational method that relies on the geometrical distribution of the three tags. Our results show that the conformation of the small yeast chromosome 3 is mating type specific in G1. Differential folding of the left arm of this chromosome can be attributed to a small DNA element which could explain why loci on this arm may be excluded from recombination with the *MAT* locus on the right arm of chromosome 3. Chromatin structural properties altered in the absence of the Asf1 histone chaperone contribute to the lineage specific chromosome organization and the relative position of the three mating type loci.

## Introduction

The three-dimensional organization of the genome was shown to dynamically adapt to nuclear function and complexity [1–3]. Chromatin fibers can be represented by a polymer random coil adopting a considerable, largely unappreciated number of states in response to DNA metabolism [4–7]. How the intrinsic folding of a chromosome within the nucleus and the relative position of loci on specific chromosomes contribute to these processes is not known. Chromosome conformation capture techniques that rely on protein-DNA cross-linking have provided precious information on the frequency of three-dimensional long range molecular contacts between genomic DNA segments [4,8–10]. Microscopy approaches are, on the other hand, necessary to further our understanding of the dynamics of DNA transactions because they allow analysis of live and single cells [11,12]. Using two-color distance measurements between two fluorescently labelled loci in 3D in fixed or living cells has yielded data to infer chromatin compaction parameters by polymer modeling [13–15]. These parameters were also used to model 4C and HiC data (for example see [4,8,12]) or to simulate genome-wide chromosome organization[2]. Because the nucleosome fiber is highly flexible, inferring fiber properties from measuring distances between two labeled loci implies making a number of assumptions to describe the actual path of the fiber. In order to obtain spatial, 3D information, at least 3 points in space are needed. Three points provide geometrical information that can be used to establish physical models. In the nucleus, these points can include reference structures, such as the nuclear envelope or the nucleolus in yeast, or three distinct DNA tags. Few previous studies used three labeled loci: in fixed, mammalian cells, distances and angles within a triangle formed by three probes in the same nucleus were measured to study changes in chromatin domain compaction [16]; in live bacteria cells, three labels were used to determine the position of two chromosomal loci relative to a third, reference one, in 2D [17]. A geometrical interpretation of changes in chromosome folding was not proposed.

The challenge for the analysis of three points in space is to develop mathematical algorithms allowing comparison of two sets of data, because commonly used tests for comparing distributions (1D, using Wilcoxon or KS tests) are no longer applicable. New computational tools are needed to identify specific features which may not be the most prominent ones, notably in cellular systems evolving over time or in space. To address this need, we developed a new system to fluorescently label three distinct genomic loci in living cells simultaneously and implemented mathematical algorithms to analyze the relative 3D positions of the labelled DNA loci.

We used *S*. *cerevisiae* chromosome 3 (Chr3) as a model to study its folding. Chr3 is a short chromosome of only 320 kb with a tripartite organization: two AT rich domains flank a GC-rich centromere proximal region and forms a ring-like structure mediated by frequent contacts between heterochromatin loci near the ends [18–20]. This chromosome has received particular attention because the study of the mating type loci, and their interconversion, contributed to fundamental knowledge about cell lineage control, silencing and recombination [21]. Numerous genetic data contributed to a better understanding of the directionality of the mating type switch, yet the underlying mechanism is not known [22,23]. A role for chromosome architecture is usually invoked as a possible driving force for donor choice [24–27]. We have the possibility now to address this question by studying the nuclear position of the three mating type loci in the nucleus simultaneously.

In this study, we adapted a third system, based on the λ operator[28], from bacteria to yeast. In combination with the widely used Tet and Lac operators, we simultaneously tag three distinct loci in live yeast cells. We then developed a computational approach to demonstrate, that the frequency of specific positional combinations between three loci point to folding properties of a small chromosome. We find that folding of the left arm is different in *MAT* **a** and *MAT*α cells. In strains lacking either a component involved in chromatin compaction, the chaperone Asf1, or the recombination enhancer (RE), the mating type specific conformation of Chr3 is altered. Our results suggest that chromosomal organization brought about by fiber folding and heterochromatic domains contribute to control of the yeast mating type loci by regulating long-range contacts.

## Methods

### Lambda cloning and strain construction

**Fig 1:**
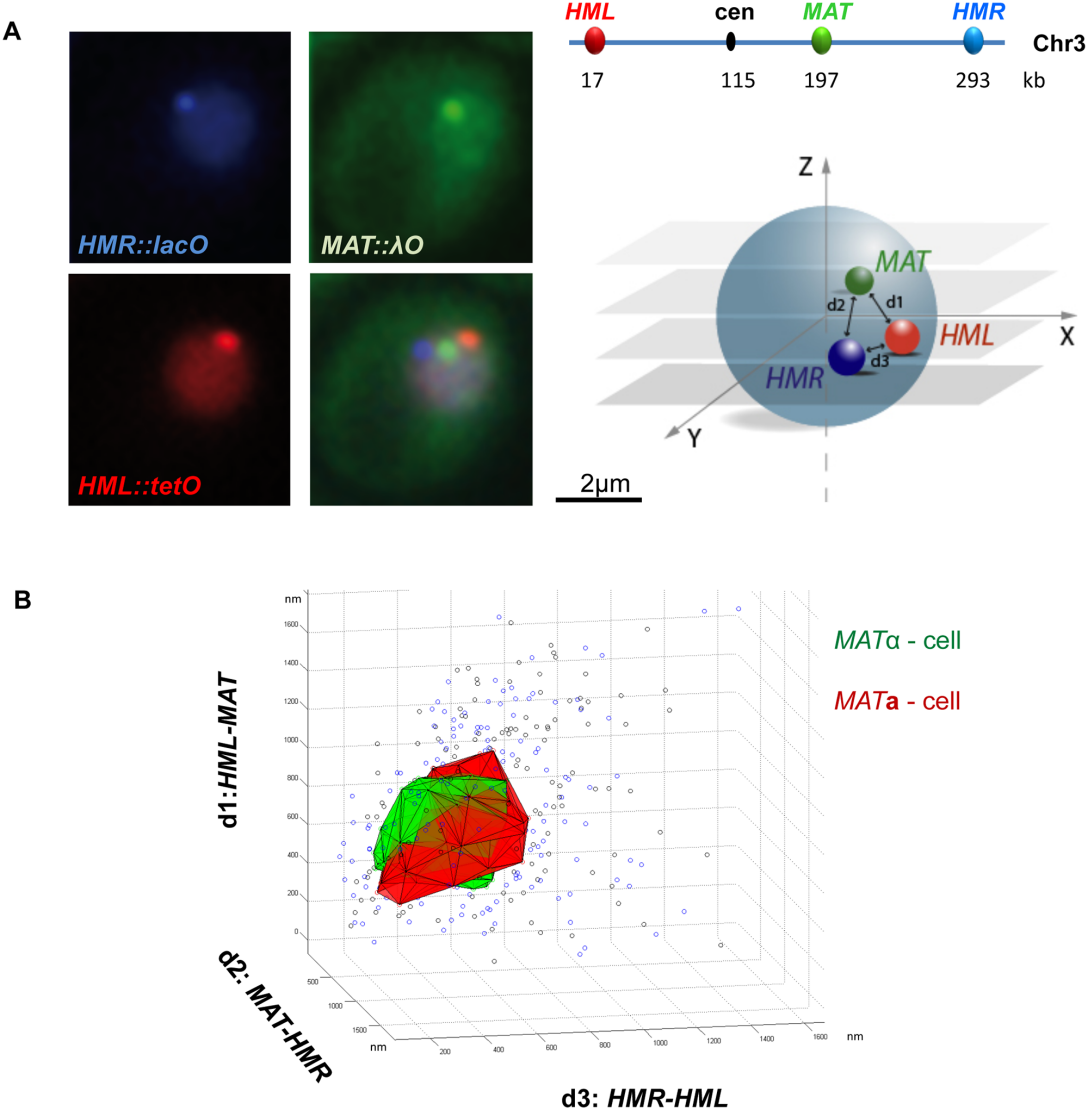
Cell type specific spatial organization of the three mating type loci on yeast chromosome 3. A) Representative wide-field fluorescent images of the the mRFP-tetR, λcI-YFP and CFP-lacI foci at *HML*, *MAT and HMR*. Insertion sites of the TetO, λO and LacO arrays on S.c. Chr3 are shown. B) Combinations of the geometric coordinates d1, d2, d3 are plotted for each nucleus in *MAT* **a** (black dots) and *MAT*α (blue dots). The 50% most frequent combinations in *MAT* **a** (red) and *MAT*α (green) are included in a 3D volume.

To simultaneously label three distinct loci, we adapted the bacterial λ repressor operator system (λO and λCi) for use in yeast [28,29] and combined it with the existing Lac and Tet systems [30](Fig 1A). Strains bearing three distinct labels were created by integration of constructs encoding fluorescent repressor fusion proteins followed by the integration of the operator sequences into the yeast genome by transformation. Yeast strains are listed in Table 1. Expression of an operon containing the phage λcI repressor gene fused to the gene encoding YFP was placed under the control of the pURA3 promoter. The λ repressor sequence bears amino acid modifications G48S and Y210H which strengthen DNA binding and reduce tetramerization, respectively [28]. The λcI sequence was amplified by PCR using pRFG116 and kindly provided by Dr D Chattoraj. During the PCR reaction a NotI recognition site was introduced. The pURA3 promoter was amplified from the pCJ97 plasmid with an introduction of a NheI site. The two fragments were then digested by NheI and ligated to generate a pURA3- λcI fragment. This fragment was double-digested with EcoRI and NotI, and ligated to the double-digested (EcoRI/NotI) pCJ97 plasmid bearing a YFP sequence.

**Table 1:** Yeast strains used in this study.

Name	Genotype
yIL02	*Mat* **a** *ade2-1 can1-100 his3-11*, *15 leu2-3*, *112 trp1-1 ura3-1 ade2-1*::*His3p-CFP-lacI-URA3p-λcI-YFP-ADE2*, *TetR-mRFP*:*NAT1*
yIL03	*Mat*α *ade2-1 can1-100 his3-11*, *15 leu2-3*, *112 trp1-1 ura3-1 ade2-1*::*His3p-CFP-lacI-URA3p-λcI- YFP-ADE2*, *TetR-mRFP*:*NAT1*
yIL30	*Mat* **a** *ade2-1 can1-100 his3-11*, *15 leu2-3*, *112 trp1-1 ura3-1 ade2-1*::*His3p-CFP-lacI-URA3p-λcI-*
	*YFP-ADE2*, *TetR-mRFP*:*NAT1*, *MAT5'*:: *λO-HIS*, *HMR*:: *LacO-TRP*, *HML*::*TetO-LEU*
yIL31	*Mat*α *ade2-1 can1-100 his3-11*, *15 leu2-3*, *112 trp1-1 ura3-1 ade2-1*::*His3p-CFP-lacI-URA3p-λcI- YFP-ADE2*, *TetR-mRFP*:*NAT1*, *MAT5'*:: *λO-HIS*, *HMR*:: *LacO-TRP*, *HML*::*TetO-LEU*
yIL30 *asf1*	*Mat* **a** *ade2-1 can1-100 his3-11*, *15 leu2-3*, *112 trp1-1 ura3-1 ade2-1*::*His3p-CFP-lacI-URA3p-λcI- YFP-ADE2*, *TetR-mRFP*:*NAT1*, *MAT5'*:: *λO-HIS*, *HMR*:: *LacO-TRP*, *HML*::*TetO-LEU*, *asf1*::*HYG*
yIL31 *asf1*	*Mat*α *ade2-1 can1-100 his3-11*, *15 leu2-3*, *112 trp1-1 ura3-1 ade2-1*::*His3p-CFP-lacI-URA3p-λcI- YFP-ADE2*, *TetR-mRFP*:*NAT1*, *MAT5'*:: *λO-HIS*, *HMR*:: *LacO-TRP*, *HML*::*TetO-LEU*, *asf1*::*HYG*
yIL30 Δ*re*	*Mat* **a** *ade2-1 can1-100 his3-11*, *15 leu2-3*, *112 trp1-1 ura3-1 ade2-1*::*His3p-CFP-lacI-URA3p-λcI- YFP-ADE2*, *TetR-mRFP*:*NAT1*, *MAT5'*:: *λO-HIS*, *HMR*:: *LacO-TRP*, *HML*::*TetO-LEU*, *re*::*HYG*
yIL31Δ*re*	*Mat*α *ade2-1 can1-100 his3-11*, *15 leu2-3*, *112 trp1-1 ura3-1 ade2-1*::*His3p-CFP-lacI-URA3p-λcI- YFP-ADE2*, *TetR-mRFP*:*NAT1*, *MAT5'*:: *λO-HIS*, *HMR*:: *LacO-TRP*, *HML*::*TetO-LEU*, *re*::*HYG*
yIL32	*Mat* **a** *ade2-1 can1-100 his3-11*, *15 leu2-3*, *112 trp1-1 ura3-1*, *ade2-1*::*His3p-CFP-lacI-URA3p-λcI-*
	*YFP-ADE2*, *MAT5'*:: *λO-HIS*, *TetR-mRFP-NAT*, *HML*::*LacO-TRP*, *Leu*::*TetO-LEU*
yIL33	*Mat*α *ade2-1 can1-100 his3-11*, *15 leu2-3*, *112 trp1-1 ura3-1*, *ade2-1*::*His3p-CFP-lacI-URA3p-λcI-*
	*YFP-ADE2*, *MAT5'*:: *λO-HIS*, *TetR-mRFP-NAT*, *HML*::*LacO-TRP*, *Leu*::*TetO-LEU*
yIL34	*Mat* **a** *ade2-1 can1-100 his3-11*, *15 leu2-3*, *112 trp1-1 ura3-1*, *ade2-1*::*His3p-CFP-lacI-URA3p-λcI-*
	*YFP-ADE2*, *MAT5'*:: *λO-HIS*, *TetR-mRFP-NAT*, *ARS1413*::*LacO-TRP*, *VI-L*:: *TetO-LEU*
yIL35	*Mat*α *ade2-1 can1-100 his3-11*, *15 leu2-3*, *112 trp1-1 ura3-1*, *ade2-1*::*His3p-CFP-lacI-URA3p-λcI- YFP-ADE2*, *MAT5'*:: *λO-HIS*, *TetR-mRFP-NAT*, *ARS1413*::*LacO-TRP*, *VI-L*:: *TetO-LEU*

Following the validation steps discussed above, we subcloned the pURA3- λcI-YFP in a plasmid bearing pHIS-CFP-lacI-pUra3-TetR-YFP (named pGVH30 [18]), plasmid which allows expression of two fusion proteins from a single plasmid thus requiring a unique selection marker (ADE2), finally leading to plasmid pIL01. The λO were extracted from the bacterial pRFB122 plasmid by an XhoI digestion and ligated to the pSR6 plasmid [31] and digested by XhoI/SalI. Integration of the operator repeats was performed using a cloning free technique. The method entails insertion of a marker gene generated by PCR using long primers, with the optimal size of the locus-specific primer tails varying from 60 to 80 nt, near the locus of interest. The marker is then replaced by the operator repeats found in pSR plasmids. The following PCR-amplified genomic fragments (SGD coordinates) were used for insertion within 0.5 to 4 kb from the respective loci: 15160 kb to 15773 kb for *HML*, 294898 kb to 295245 kb for *HMR*, 90917 kb to 92521 kb for *LEU*, 239254 kb to 240927 kb for *ARS1413* and 16431 kb to 17993-kb for *TelVI*. The PCR-amplified sequences of *HMR* and *HML* were cloned into a pAFS52-lacO plasmid and in the pAFS59-tetO bearing plasmid, respectively[18]. The lambda operator repeats were integrated at 197197–197310 kb on Chr3 to label the *MAT* locus. The *ASF1* gene (196334 kb to 197080 kb on Chr10) or the RE (29083 kb to 29748 kb on Chr3) were replaced by the hygromycin resistance gene amplified from pAG32 (1743bp). Alternatively, the RE (28987–29852 on Chr3) was replaced by loxP flanked hygromycin resistance gene amplified from pGI10. Galactose induced expression of the recombinase from pHS47 induces deletion of the entire cassette (162bp remaining plasmid sequences). The lambda operator (λO) system comprises a relatively small number of repeated binding sites; 64 repeats compared to the usual 128–256 repeats of TetO and LacO arrays. The focus formed by binding of multiple repressor fusion proteins to the arrays integrated into the genome is easily detectable using conventional fluorescence microscopy (Fig 1A). The constitutive expression of the λcI repressor fused to YFP and its binding to a short 1.5kb DNA λO fragment was not toxic to the cell, which displayed growth rates identical to unmodified yeast cultures. λcI-YFP fusion proteins diffuse freely in the cytoplasm, and in the nucleoplasm apart from the vacuole (Fig 1A). The focus formed at a site near the *MAT* locus (197 kb along right arm of Chr3) tagged using λO was positioned in the center of the nuclear lumen with the same frequency as the same genetic locus tagged with LacO.

### Microscopy and image analysis

Live microscopy was performed using an Olympus IX-81 wide-field fluorescence microscope, equipped with a CoolSNAPHQ camera (Roper Scientific) and a Polychrome V (Till Photonics), electric piezo with accuracy of 10 nm and imaged through an Olympus oil immersion objective 100X PLANAPO NA1.4. Yeast cells were spread on a concave microscopy slide filled with SD-agarose (YNB + 2% sugar/ carbon source + 3%(w/v) agarose). Acquisition of CFP, mRFP and YFP was performed in 3D (21 focal planes at 0.2 μm distance intervals; 500 ms, 300 ms, 500 ms acquisition times respectively). Fluorescent intensities of the acquired spots were identical in both cell types demonstrating that the inserted operator arrays maintained the same size. The x, y and z coordinates for each focus were automatically measured using the “Spot distance”plug in on image J (http://bigwww.epfl.ch/sage/soft/spotdistance/; D Sage, EPFL; [29]. This program uses multi-channel z-stack images to localize the center of the nucleus based on the background fluorescence of the CFP-lacI. The position of each focus is assigned to the center of gravity of the fluorescence around the brightest pixel found in this nucleus in a filtered version of the image. The signals are scored on 3D stacks using at least 200 nuclei, monitoring nuclear integrity and cell cycle stage through bud shape and nuclear diameter. Distances between pairs of loci can be determined in 3D using this Image J plugin. Statistical analysis was performed using the Wilcoxon test.

### Mating type switching assay

Expression of the HO-endonuclease gene from the pHO (URA selection[32]) was induced by addition of 2% filtered galactose (SIGMA) to a yeast culture exponentially growing in 2% raffinose. For quantitative PCR reactions, Bio-Rad SybrGreen Supermix was used in the presence of 30ng of genomic DNA and 0.26μM of each primer. PCR were run on ABI 7900. The *MAT* **a**-specific primers were 5’-GGCATTACTCCACTTCAAGT (P1) and 5′-ATGTGAACCGCATGGGCAGT (P2). The MAT*α*-specific primers were 5′-ATGTGAACCGCATGGGCAGT (P2) and 5′- GCAGCACGGAATATGGGACT (P3). Primers specific for the *ARG5*, *6* locus 5′- CAAGGATCCAGCAAAGTTGGGTGAAGTATGGTA and 5′- GAAGGATCCAAATTTGTCTAGTGTGGGAACG or for the actin locus were used for normalization. All qPCR assays were accompanied by reactions using dilutions of genomic DNA from wt strains’ 0h input sample to assess the linearity of the PCR signal and to create calibration curves.

### Modeling experience and problem formulation

One considers a fiber with *N* nodes. Each state ***E***_***k***_ of this fiber defined in a reference frame R_k_ is represented by *N* nodes:

$${\left({\text{x}}_{1},{\text{y}}_{1},{\text{z}}_{1}\right)}_{{\text{R}}_{\text{k}}},{\left({\text{x}}_{2},{\text{y}}_{2},{\text{z}}_{2}\right)}_{{\text{R}}_{\text{k}}},\;...\;\;,\;{\left({\text{x}}_{\text{N}},{\text{y}}_{\text{N}},{\text{z}}_{\text{N}}\right)}_{{\text{R}}_{\text{k}}}k=1...M$$

For all states $E_{k_{1}}$ and $E_{k_{2}},\;R_{k_{1}}\neq R_{k_{2}}$ since k_1_≠k_2_.

On a chromosome represented as a polymer fiber, the center of gravity of each fluorescently labelled locus represents a node. *N* loci can be represented by (x_i_, y_i_, z_i_)_i = 1…N_.

If we denote θ_i_ the angle around the node i.

$${\text{d}}_{\text{i}}^{1}=\{\begin{array}{c} {\text{d}}_{\text{i},\text{i}+1}\;\text{if}\;\text{i}<\text{N}\\ {\text{d}}_{\text{N},1}\;\text{if}\;\text{i}=\text{N} \end{array}$$ (1)

$${\text{d}}_{\text{i}}^{2}=\{\begin{array}{c} {\text{d}}_{\text{i}-1,\text{i}}\;\text{if}\;\text{i}>1\\ {\text{d}}_{\text{N},1}\;\text{if}\;\text{i}=1 \end{array}$$ (2)

For each local analysis around node *i*, we make the following change of variables:

$$\left(x_{i-1},y_{i-1},z_{i-1},x_{i},y_{i},z_{i},x_{i+1},y_{i+1},z_{i+1}\right)≻\left(d_{i}^{1},d_{i}^{2},\theta_{i}\right)$$

The main advantage of these new variables is their invariance during the reference frame change. To analyze the variations of positions around the node *i*, we introduce the normalized principal components analysis operator $\underset{norm}{PCA}$ and consider a set of experimental data ${\left\{{\left(d_{i}^{1,k},d_{i}^{2,k},\theta_{i}^{k}\right)}_{i\;=\;1..N}\right\}}_{k\;=\;1..M}$ acquired around all nodes *i* in all nuclei of state E_k_. We denote by C_i_ and P_i_ the correlation matrix of the principal component analysis and the diagonal matrix of eigenvalues.

$$\left[{\text{C}}_{\text{i}},{\text{P}}_{\text{i}}\right]=\underset{\text{norm}}{\text{PCA}}{\left\{\left({\text{d}}_{\text{i}}^{1,\text{k}},{\text{d}}_{\text{i}}^{2,\text{k}},\theta_{\text{i}}^{\text{k}}\right)\right\}}_{\text{k}=1..\text{M}}$$ (3)

For all nuclei, we can write the coordinates of red, green and blue loci in the referential linked to the microscope as ${\left(x_{r},y_{r},z_{r}\right)}_{R_{microscope}},{\left(x_{g},y_{g},z_{g}\right)}_{R_{microscope}}$. Taking the red locus as the origin, we can write

$$\begin{array}{l} {\left({\text{x}}_{\text{r}},{\text{y}}_{\text{r}},{\text{z}}_{\text{r}}\right)}_{{\text{R}}_{\text{microscope}}}\equiv {\left(0,0,0\right)}_{{\text{R}}_{\text{microscopenuclei}}}\;,\;\\ {\left({\text{x}}_{\text{g}},{\text{y}}_{\text{g}},{\text{z}}_{\text{g}}\right)}_{{\text{R}}_{\text{microscope}}}\equiv {\left({\text{x}}_{\text{g}}-{\text{x}}_{\text{r}},{\text{y}}_{\text{g}}-{\text{y}}_{\text{r}},{\text{z}}_{\text{g}}-{\text{z}}_{\text{r}}\right)}_{{\text{R}}_{\text{microscopenuclei}}}\;,\;\\ {\left({\text{x}}_{\text{b}},{\text{y}}_{\text{b}},{\text{z}}_{\text{b}}\right)}_{{\text{R}}_{\text{microscope}}}\equiv {\left({\text{x}}_{\text{g}}-{\text{x}}_{\text{r}},{\text{y}}_{\text{g}}-{\text{y}}_{\text{r}},{\text{z}}_{\text{g}}-{\text{z}}_{\text{r}}\right)}_{{\text{R}}_{\text{microscopenuclei}}}. \end{array}$$

Referential R_microscopenuclei_ is called pseudo-referential because its origin is inside the nucleus and its basis vectors are linked to the microscope. To overcome the orientation problem inherent to the nuclear sphericity, we can define new variables simple enough to analyze the position of the three loci as (1 and 1) (S1 Fig). Let us take:

$${\text{d}}_{1}={\left[{\left({\text{x}}_{\text{g}}-{\text{x}}_{\text{r}}\right)}^{2}+{\left({\text{y}}_{\text{g}}-{\text{y}}_{\text{r}}\right)}^{2}+{\left({\text{z}}_{\text{g}}-{\text{z}}_{\text{r}}\right)}^{2}\right]}^{\frac{1}{2}}$$ (4)

$${\text{d}}_{2}={\left[{\left({\text{x}}_{\text{b}}-{\text{x}}_{\text{r}}\right)}^{2}+{\left({\text{y}}_{\text{b}}-{\text{y}}_{\text{r}}\right)}^{2}+{\left({\text{z}}_{\text{b}}-{\text{z}}_{\text{r}}\right)}^{2}\right]}^{\frac{1}{2}}$$ (5)

and

$$\theta =\text{arccos}\left(\frac{\left({\text{x}}_{\text{g}}-{\text{x}}_{\text{r}},{\text{y}}_{\text{g}}-{\text{y}}_{\text{r}},{\text{z}}_{\text{g}}-{\text{z}}_{\text{r}}\right)\circ \left({\text{x}}_{\text{b}}-{\text{x}}_{\text{r}},{\text{y}}_{\text{b}}-{\text{y}}_{\text{r}},{\text{z}}_{\text{b}}-{\text{z}}_{\text{r}}\right)}{{\text{d}}_{1}\cdot {\text{d}}_{2}}\right)$$ (6)

where ∘ is the scalar product.

With these new variables each nucleus is represented by three variables instead of nine: (x_r_, y_r_, z_r_, x_g_, y_g_, z_g_, x_b_, y_b_, z_b_)>(d_1_, d_2_, θ)

### Variables and referential

#### Projection

**Fig 2:**
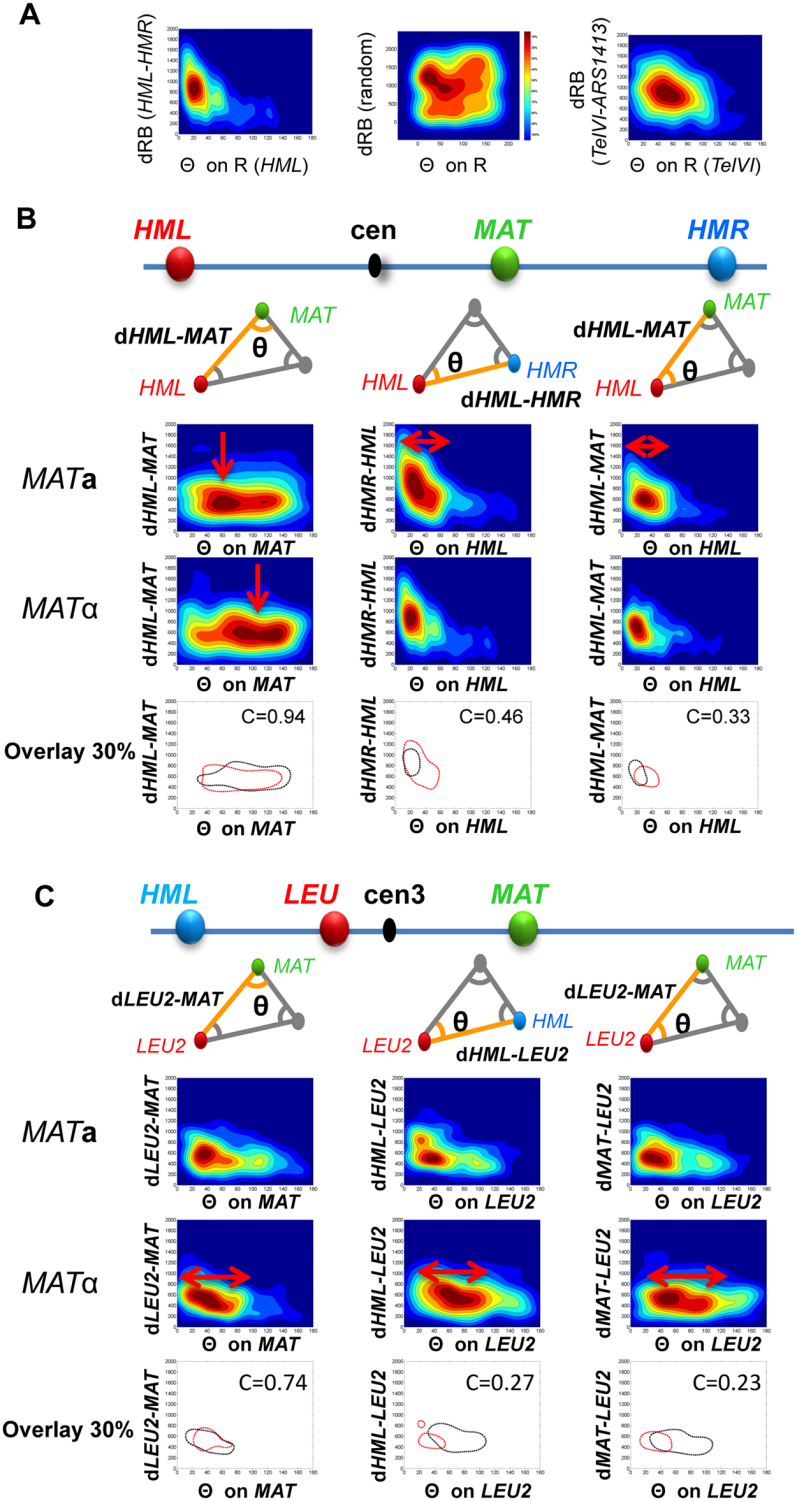
Folding of the left arm of the chromosome 3 differs in a subset of *MAT*a and *MAT*α cells. A) 3D data are projected onto a unique 2D plane (eg. d1/θ; 9 projections can be generated from each data set. Density maps (warm colors for high density and cold colors for weak density in 10% increments) are generated for each projection in 2D. Examples shown are density maps where the origin is set to the red spot (R), the d1 distances (vector RB red to blue) are aligned and plotted relative to the angle θ of the RGB triangle at R. left panel: R = *HML*, d1 = RB = *HML*—*HMR*), θ at *HML*. Center panel: Example of a density map obtained from a simulation based on a random draw of relative positions between 3 loci. Right panel: *TelVI* = R, *ARS*1413 = B and *MAT*. B-C) *HML*, *MAT* and *HMR* (B; YIL30/YIL31; n = 223 and 276)) or *LEU*, *MAT* and *HML* (C; YIL32/33; n = 409 and 323) were labelled using TetO/mRFP-tetR, λO/ λcI-YFP and LacO/CFP-lacI respectively. Examples of density maps are shown to compare the distribution between the 3 loci in *MAT* **a** and *MAT*α cells. Red arrows highlight changes between *MAT* **a** and *MAT*α cells. An overlay of the contours of the density area representing 30% of the analyzed *MAT* **a** (black) and *MAT* α (red) is represented. The correlation factor c is given for 30% contour. Complete sets of density maps are shown in S2–S3 Figs.

Each nucleus is represented by the three components (d_1_, d_2_, θ). We project all points ${\left(d_{1}^{i},d_{2}^{i},\theta^{i}\right)}_{i\;=\;1..M}$ in (d_1_, d_2_), (θ, d_1_) and (θ, d_2_) planes (Fig 2A and S1 Fig), $\left(X_{i},X_{j}\right)\equiv {\left(X_{i},X_{j}\right)}_{X_{i}\;=\;\theta ,d_{1},d_{2}\text{ / }X_{j}\;=\;\theta ,d_{1},d_{2}\text{ / }i\neq j}$. In each plane, the probability density function is estimated by Parzen-Rozenblatt method [33].

#### Kernel density estimation

In the Parzen-Rozenblatt methods, estimations lie in the choice of the kernel **K**. We use a Gaussian kernel[34]. For x,y in **R** ^**2**^, the kernel **K** is given by:

$$\text{K}\left(\text{x},\text{y},\text{t}\right)=\frac{1}{2\pi \text{t}}{\text{e}}^{\frac{-{\left(\text{x}-\text{y}\right)}^{\text{t}}\left(\text{x}-\text{y}\right)}{2\text{t}}}$$ (7)

This Kernel allows us to compute the probability of the density function *f*. The cumulative distribution function *F(x*, *y)* is plotted within ten regions defined as

$${\text{U}}_{\text{k}}=\left\{\left(\text{x},\text{y}\right)\text{ / F}\left(\text{x},\text{y}\right)⩽\frac{\text{k}}{10}\right\}$$ (8).

Each density map (X_i_,X_j_) is composed of ten levels $U_{k}\;=\;{\left(X_{i}^{kl},X_{j}^{kl}\right)}_{k\;=\;1..10\text{ / }l\;=\;1.\;..card\left(X_{i}^{k}\right)}$ where $card\left(X_{i}^{k}\right)\;$ is the number of nuclei inside level *k*, giving the relation U_1_⊂U_2_⊂…⊂U_10_

For each map U_k_, we denote $\mu_{k}\;=\;\left(\mu_{k}^{i},\mu_{k}^{j}\right)$ the mean vector and $\sigma_{k}\;=\;\left(\sigma_{k}^{i},\sigma_{k}^{j}\right)$ the standard deviation vector.

$$\mu_{\text{k}}^{\text{i}}=\frac{\sum_{\text{l}=1..\text{card}\left({\text{X}}_{\text{i}}^{\text{k}}\right)}{\text{X}}_{\text{i}}^{\text{kl}}}{\text{card}\left({\text{X}}_{\text{i}}^{\text{k}}\right)}$$ (9)

$$\mu_{\text{k}}^{\text{j}}=\frac{\sum_{\text{l}=1..\text{card}\left({\text{X}}_{\text{j}}^{\text{k}}\right)}{\text{X}}_{\text{j}}^{\text{kl}}}{\text{card}\left({\text{X}}_{\text{j}}^{\text{k}}\right)}$$ (10)

$$\sigma_{\text{k}}^{\text{i}}=\sqrt{\frac{\sum_{\text{l}=\text{card}\left({\text{X}}_{\text{i}}^{\text{k}}\right)}{\left({\text{X}}_{\text{i}}^{\text{kl}}-\mu_{\text{k}}^{\text{i}}\right)}^{2}}{\text{card}\left({\text{X}}_{\text{i}}^{\text{k}}\right)}}$$ (11)

$$\sigma_{\text{k}}^{\text{j}}=\sqrt{\frac{\sum_{\text{l}=\text{card}\left({\text{X}}_{\text{j}}^{\text{k}}\right)}{\left({\text{X}}_{\text{j}}^{\text{kl}}-\mu_{\text{k}}^{\text{j}}\right)}^{2}}{\text{card}\left({\text{X}}_{\text{j}}^{\text{k}}\right)}}$$ (12)

Let us take two different experiments **A and B**, and (U_k_)_k = 1…10_ level intensity associated to **A** and (V_k_)_k = 1…10_ level intensity associated to **B**. We denote $\mu_{k_{U}}$ and $\mu_{k_{V}}$ associated mean vectors, $\sigma_{k_{U}}$ and $\sigma_{k_{V}}$ associated standard deviation vectors at the level *k*. For two levels, k_1_ in the **A**-experiment and k_2_ in the **B**-experiment we define

$$\rho_{{\text{k}}_{1}{\text{k}}_{2}}^{1\text{ij}}=\frac{\text{min}\left(\mu_{{\text{k}}_{\text{U}}}^{\text{i}},\mu_{{\text{k}}_{\text{V}}}^{\text{i}}\right)}{\text{max}\left(\mu_{{\text{k}}_{\text{U}}}^{\text{i}},\mu_{{\text{k}}_{\text{V}}}^{\text{i}}\right)}\frac{\text{min}\left(\mu_{{\text{k}}_{\text{U}}}^{\text{j}},\mu_{{\text{k}}_{\text{V}}}^{\text{j}}\right)}{\text{max}\left(\mu_{{\text{k}}_{\text{U}}}^{\text{j}},\mu_{{\text{k}}_{\text{V}}}^{\text{j}}\right)}$$ (13)

and

$$\rho_{{\text{k}}_{1}{\text{k}}_{2}}^{2\text{ij}}=\frac{\text{min}\left(\mu_{{\text{k}}_{\text{U}}}^{\text{i}},\mu_{{\text{k}}_{\text{V}}}^{\text{i}}\right)}{\text{max}\left(\mu_{{\text{k}}_{\text{U}}}^{\text{i}},\mu_{{\text{k}}_{\text{V}}}^{\text{i}}\right)}\frac{\text{min}\left(\mu_{{\text{k}}_{\text{U}}}^{\text{j}},\mu_{{\text{k}}_{\text{V}}}^{\text{j}}\right)}{\text{max}\left(\mu_{{\text{k}}_{\text{U}}}^{\text{j}},\mu_{{\text{k}}_{\text{V}}}^{\text{j}}\right)}\frac{\text{min}\left(\sigma_{{\text{k}}_{\text{U}}}^{\text{i}},\sigma_{{\text{k}}_{\text{V}}}^{\text{i}}\right)}{\text{max}\left(\sigma_{{\text{k}}_{\text{U}}}^{\text{i}},\sigma_{{\text{k}}_{\text{V}}}^{\text{i}}\right)}\frac{\text{min}\left(\sigma_{{\text{k}}_{\text{U}}}^{\text{j}},\sigma_{{\text{k}}_{\text{V}}}^{\text{j}}\right)}{\text{max}\left(\sigma_{{\text{k}}_{\text{U}}}^{\text{j}},\sigma_{{\text{k}}_{\text{V}}}^{\text{j}}\right)}$$ (14)

$\rho_{k_{1}k_{2}}^{1ij}$ is a correlation in first approximation and $\rho_{k_{1}k_{2}}^{2ij}$ is a correlation in second approximation with $\rho_{k_{1}k_{2}}^{1ij}\geq \rho_{k_{1}k_{2}}^{2ij}$.

### Inverse problem and abstract model

**Fig 3:**
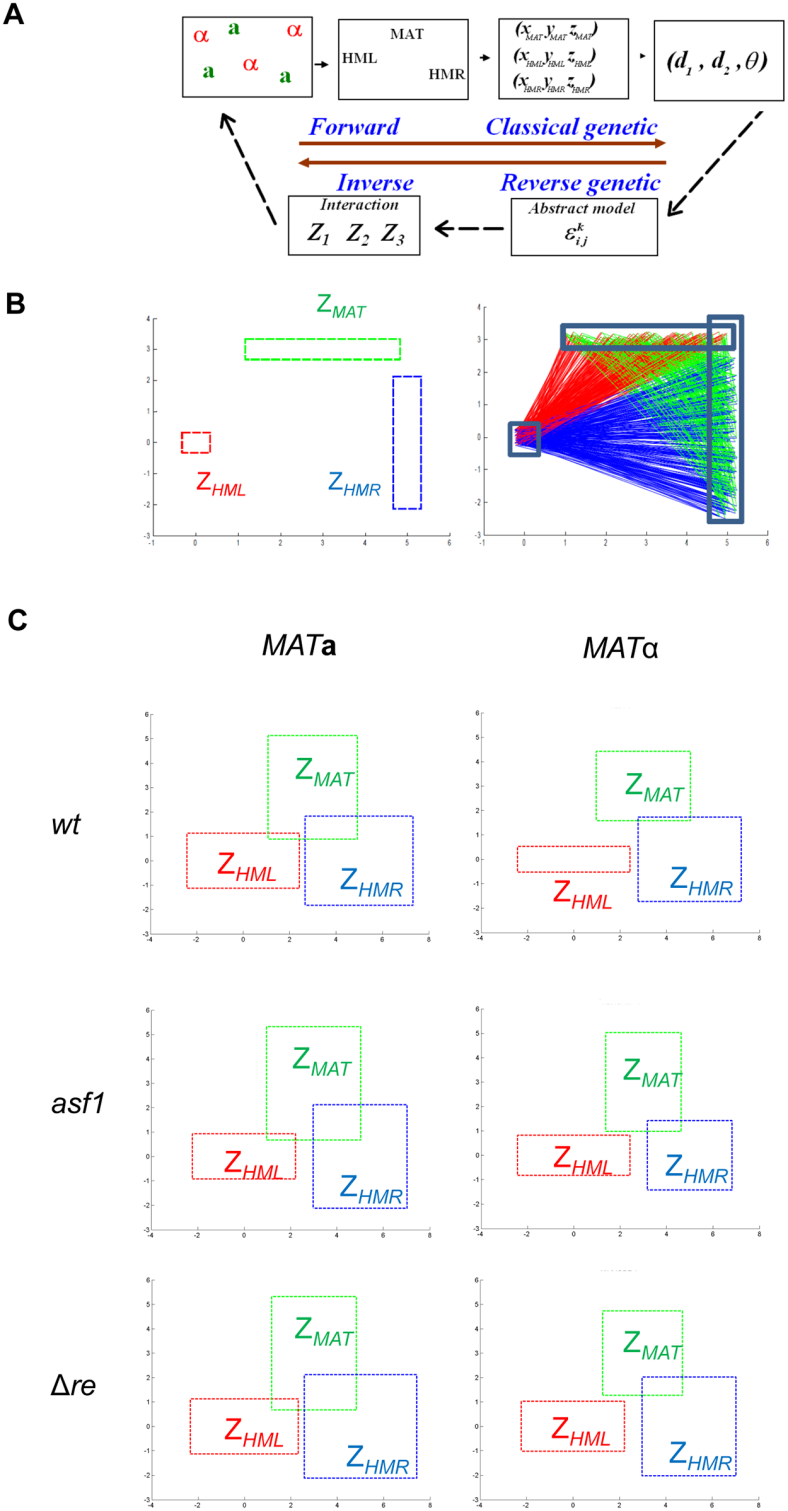
An abstract model to determine the relative physical constraint of three linked loci in wt and mutant strains. A) The forward mathematical approach allows determining features of a subpopulation of relative positions between 3 loci in space. The inverse computational approach consists in modeling the relative positions or survival zones of loci whose positions are spatially linked. The underlying polymer fiber imposes constraints on the positions each locus can adapt. Iterative calculation defines the survival zones of each locus (or node) which can predict biological relevant features. B-C) Survival Zones (Z) of *HML*, *MAT* and *HMR*. The initial position of the 3 loci is set based on the estimated conformation for Chr3 (B). Iterations were run using Eq 25 (see Methods) and the statistically most significant zones are represented for wt, *asf1* mutants and strains in which the recombination enhancer element was deleted (C).

We developed an original abstract model based on the assumption that each point of a fiber (or polymer) moves in a specific area (Fig 3A). If we consider a dynamic polymer in the plane in which we can define *N* points (x_i_, y_i_)_i = 1…N_. For each point (x_i_, y_i_), we define the survival zone as the smallest closed set P^i^ where (x_i_, y_i_) takes its values during temporal fluctuations. Any configuration Co_j_ of our polymer is defined as:

$$\exists {{\text{(x}}_{\text{k}}^{\text{j}}{\text{,y}}_{\text{k}}^{\text{j}}\text{)}}_{\text{k=1}...\text{N}}\in {\text{P}}^{\text{k}}\;\;\text{/}\;{\text{Co}}_{\text{j}}{\text{=(x}}_{\text{1}}^{\text{j}}{\text{,y}}_{\text{1}}^{\text{j}}\text{)}\to {\text{(x}}_{\text{2}}^{\text{j}}{\text{,y}}_{\text{2}}^{\text{j}}\text{)}\to ...\to {\text{(x}}_{\text{N}}^{\text{j}}{\text{,y}}_{\text{N}}^{\text{j}}\text{)}$$ (15)

Let us note Z_V1_, Z_V2_, …Z_VN_ survival zones of (x_1_, y_1_), (x_2_, y_2_)and (x_N_, y_N_) defined as ℝ^2^-closed set.

$${\text{Z}}_{{\text{V}}_{1}}\left(\varepsilon_{1}^{1},\varepsilon_{2}^{1}\right)=\left[{\text{x}}_{1}-\varepsilon_{1}^{1};{\text{x}}_{1}+\varepsilon_{1}^{1}\right]\times \left[{\text{y}}_{1}-\varepsilon_{2}^{1};{\text{y}}_{1}+\varepsilon_{2}^{1}\right]$$ (16)

$${\text{Z}}_{{\text{V}}_{2}}\left(\varepsilon_{1}^{2},\varepsilon_{2}^{2}\right)=\left[{\text{x}}_{2}-\varepsilon_{1}^{2};{\text{x}}_{2}+\varepsilon_{1}^{2}\right]\times \left[{\text{y}}_{2}-\varepsilon_{2}^{2};{\text{y}}_{2}+\varepsilon_{2}^{2}\right]$$ (17)

$${\text{Z}}_{{\text{V}}_{\text{N}}}\left(\varepsilon_{1}^{\text{N}},\varepsilon_{2}^{\text{N}}\right)=\left[{\text{x}}_{\text{N}}-\varepsilon_{1}^{\text{N}};{\text{x}}_{\text{N}}+\varepsilon_{1}^{\text{N}}\right]\times \left[{\text{y}}_{\text{N}}-\varepsilon_{2}^{\text{N}};{\text{y}}_{\text{N}}+\varepsilon_{2}^{\text{N}}\right]$$ (18)

We can define an abstract polymer from the knowledge of the *N* survival zones. Here, the abstract fiber will be close to the real fiber as soon as the survival zones will be small and the number of nodes is large enough. We can build several random configurations of our polymer knowing the survival zones.

$${\left({\text{X}}_{1,\text{i}},{\text{Y}}_{1,\text{i}}\right)}_{\text{i}=1...\text{K}}=\text{ rand}\left({\text{Z}}_{{\text{V}}_{1}}\right)$$ (19)

$${\left({\text{X}}_{2,\text{i}},{\text{Y}}_{2,\text{i}}\right)}_{\text{i}=1...\text{K}}=\text{ rand}\left({\text{Z}}_{{\text{V}}_{2}}\right)$$ (20)

$${\left({\text{X}}_{\text{N},\text{i}},{\text{Y}}_{\text{N},\text{i}}\right)}_{\text{i}=1...\text{K}}=\text{ rand}\left({\text{Z}}_{{\text{V}}_{\text{N}}}\right)$$ (21)

where $rand\left(Z_{V_{i}}\right)$ denotes the unform random function in the set $Z_{V_{i}}$

We can construct an abstract configuration:

$${\text{Co}}_{\text{i}}^{\text{a}}=\left({\text{X}}_{1,\text{i}},{\text{Y}}_{1,\text{i}}\right)\to \left({\text{X}}_{2,\text{i}},{\text{Y}}_{2,\text{i}}\right)\to ...\to \left({\text{X}}_{\text{N},\text{i}},{\text{Y}}_{\text{N},\text{i}}\right)$$ (22)

For the given abstract configuration set, we can define as (1 and 2) new system variables:

$${\left\{{\left({\text{D}}_{\text{i},\text{k}}^{1},{\text{D}}_{\text{i},\text{k}}^{2},\Theta_{\text{i},\text{k}}\right)}_{\text{k}=1...\text{M}}\right\}}_{1...\text{N}}$$

Let us note $C_{i}^{a}$ and $P_{i}^{a}$ correlation matrix and diagonal eigenvalues matrix of the abstract system

$$\left[{\text{C}}_{\text{i}}^{\text{a}},{\text{P}}_{\text{i}}^{\text{a}}\right]=\underset{\text{norm}}{\text{PCA}}{\left\{\left({\text{D}}_{\text{i},\text{k}}^{1},{\text{D}}_{\text{i},\text{k}}^{2},\Theta_{\text{i},\text{k}}\right)\right\}}_{\text{k}=1..\text{M'}}$$ (23)

Where *M'* denotes the number of abstract configurations and *N* the number of nodes.

For a given set of points, the system of survival zones that best correlates with our experimental data has to be determined. Let us note

$${\text{M}}_{\Delta }^{\text{i}}={\text{C}}_{\text{i}}-{\text{C}}_{\text{i}}^{\text{a}}$$ (24)

as the difference between the experimental correlation matrix and abstract correlation matrix.

The optimal abstract model will be defined by:

$$\left\{\left({\text{x}}_{1},{\text{y}}_{1},\varepsilon_{1}^{1},\varepsilon_{2}^{1}\right),\left({\text{x}}_{2},{\text{y}}_{2},\varepsilon_{1}^{2},\varepsilon_{2}^{2}\right),...,\left({\text{x}}_{\text{N}},{\text{y}}_{\text{N}},\varepsilon_{1}^{\text{N}},\varepsilon_{2}^{\text{N}}\right)\right\}={\text{argmin}}_{\varepsilon_{\text{j}}^{\text{i}}\in \text{D}}\underset{}{{\sum^{}}_{\text{i}=1...\text{N}}}\;∥{\text{M}}_{\Delta }^{\text{i}}\;∥{\;}_{\text{frob}}$$ (25)

||. ||_frob_ denotes the Frobenian norm of a square matrix. *D* is the base interval.

### Inverse problem solving

Eq (25) is solved iteratively. The center of each survival zone is fixed by conservation of proportions between the three distances.

On chromosome 3, the center of *HML* positions is taken at (0,0), the center of *HMR* is fixed at (5,0). If d_HML-MAT_, d_MAT-HMR_ and d_HML-HMR_ are experimental mean distances, the lengths r_0_ and r_1_ are given by:

$$\frac{5}{{\text{d}}_{\text{HML}-\text{HMR}}}=\frac{{\text{r}}_{0}}{{\text{d}}_{\text{HML}-\text{MAT}}}=\frac{{\text{r}}_{1}}{{\text{d}}_{\text{MAT}-\text{HMR}}}$$ (26)

The center of the *MAT* zone is defined as the intersection of two circles. The circle with (0,0) center and r_0_ as radius. The second circle has (5,0) as a center and r_1_ as radius.

Iterations are made using the variables $\varepsilon_{1}^{1},\varepsilon_{2}^{1},\varepsilon_{1}^{2},\varepsilon_{2}^{2},\varepsilon_{1}^{3}$ and $\varepsilon_{2}^{3}$, in the range [0;5]. Our code has been parallelized on one hundred CPU cards using MPI (Message Passing Interface). The step of subdivision for the iterative resolution is taken at 0.2 to yield 5^12^ abstract configurations. Each experiment represents ~100 hours of calculation; computing of survival zones for all conditions tested took 1500 hours of calculation.

## Results and Discussion

### Differentiating geometric distribution of three distinct chromosome loci

Combinations of the widely used Tet and Lac repressors, each fused to a fluorophore with a distinct emission spectrum, has proven to be a valuable tool for simultaneous visualization of two loci [18,19,30,35–37]. In order to simultaneously label three distinct loci to track their relative position, we adapted a third system based on the the bacterial λ repressor operator system (λO and λCi) [19] for use in yeast (Fig 1A). Operator sequences were inserted by homologous recombination near the *HML*, *MAT* and *HMR* or near *HML*, *LEU* and *MAT* loci on Chr3. Acquisition of fluorescent RFP, YFP and CFP proteins fused to the Lac, Tet and λ repressors, respectively, was performed in 3D.

We introduce a normalized principal components analysis (PCA) operator to convert the automatically measured x, y and z coordinates for each focus into geometrical variables (see Methods). The resulting coordinates of the formed triangle, here d1 (side of the triangle delimited by *HML- MAT*), d2 (*MAT-HMR)* and d3 (*HMR-HML*), were plotted on the same graph for all analyzed cells (Fig 1B). The large variation in positional combinations of the three loci reflects the highly dynamic nature of chromosome folding in yeast [38]. The *MAT* locus is mobile [18,39,40] but the two silent mating type loci, despite their frequent juxtaposition, also change position relative to each other within 10–30 seconds [19]. 50% of the most frequent positions observed for the three loci were included in a volume whose 3D surface is colored in red for *MAT* **a** and in green for *MAT*α cells (Fig 1B). A large fraction of the d1_d2_d3 combinations are correlated in both mating types (intersection between the red and green iso-volumes). They represent the most probable conformations of Chr3 and are as such readily detected by other methods. Strikingly, subsets of relative triangular positions of the three mating type loci are specific to *MAT* **a** or *MAT*α cells. Our goal was to characterize the folding features leading to this variant part of the distribution and to correlate them with donor preference.

### Folding of the left arm of chromosome 3 is mating type specific

The distribution of angles within the triangle formed by *MAT*, *HML* and *HMR* in *MAT* **a** and *MAT*α cells was statistically significant (S1 and S2A Figs), again suggesting that certain conformations of Chr3 could be mating type specific. To extract folding features from the distribution of the three loci, we generated 2D projections of geometrical coordinates recorded for all nuclei. In the resulting density maps (Fig 2 and S2–S7 Figs), data are grouped within 10% color-coded increments of occurrence. For each dataset, nine distinct maps are generated and compared to the equivalent maps of another dataset using PCA (see Methods). Correlation coefficients (c) between duplicate experiments using the same strain were greater than 0.8 (S2C Fig; four independent experiments 223<n<559). As a control, density maps resulting from a simulation of data obtained using random positioning of three loci within a sphere of 2 μm diameter (one example is shown in Fig 2A) were significantly different from experimental data (c<0.1). Furthermore, the distribution of three independent loci (*MAT* on Chr3, the right telomere of Chr5 and ARS1412 on Chr14) did not correlate with the ones on Chr3 (c <0.6 (n = 405)). Thus, density maps obtained for the distribution of the three mating type loci on Chr3 are non-random. Strikingly, the maps obtained in **a**-cells were distinct from those in α-cells. This was surprising because the nuclear positions of individually labelled mating type loci were previously shown not to be statistically different in **a**- and α-cells[26]. Density maps differed at the 10–50% contour levels around *HML* and *MAT* (S2C Fig). The angles formed at *HML* were significantly smaller in a fraction (~30%) of α-cells compared to **a**-cells (Fig 2B) suggesting that, in α-cells, loci on the left arm of Chr3 are more confined with respect to those on the right arm. Also, in about 20% of cells, the angle at *MAT* was much smaller in **a**-cells than in α-cells (80°-140°) for a similar distribution of *HML-MAT* distances. These data expose, for the first time, that positioning of *HML* relative to *MAT* and *HMR* differs between *MAT* **a** and *MAT*α cells.

Geometric analysis of another combination of three loci provides additional detail (Fig 2C and S3 Fig). We labeled two loci, *HML* and *LEU2*, on the left arm and one, *MAT*, on the right. Density maps confirm that a portion of the left arm of Chr3 is largely compressed in *MAT* **a** cells. For example, the angle at *LEU2* formed with the vector pointing towards *HML* was, in the 30% most representative cells, significantly smaller in *MAT* **a** than in *MAT*α cells (c = 0.27). This suggests that *HML* and *LEU2* roam in a similar volume with respect to *MAT* in *MAT* **a** but not in *MAT*α cells. Increased constraint of the *LEU2* locus supports the view that the left arm, or at least a large region of it, can crumple and shorten in *MAT* **a** cells, dynamically changing between an extended conformation and a transiently more compressed one.

### Relative physical constraint characterizes differential chromosome folding

Our goal was to determine whether the linkage to a chromosome fiber contributes to the relative positions of three distinct loci. We developed an abstract polymer model to define physical constraint imposed by the fiber (Eq (25) in Methods). Each nucleus can be taken as a state of the chromosome fiber. Hence, we have up to five hundred states of our system to describe our data. In an optimization approach, we assume a known configuration of Chr3 as the initial configuration (Fig 3A) to reduce the number of parameters to be estimated. The abstract model is a model based on the assumption that each point of a fiber (or polymer) moves uniformly in a specific area or survival zone (Z). To solve Eq (25) we used an iterative method based on the variables $\varepsilon_{1}^{1},\varepsilon_{2}^{1},\varepsilon_{1}^{2},\varepsilon_{2}^{2},\varepsilon_{1}^{3}$ and $\varepsilon_{2}^{3}$, in the range [0;5]. Our code has been parallelized on one hundred CPU cards. The step of subdivision for the iterative resolution is taken at 0.2 to yield 5.10^12^ abstract configurations. We can thus determine interactions between the survival zones Z_MAT_, Z_HML_ and Z_HMR_ as the extent by which two points roam in the same space while being under the physical constraint of the fiber. Z_MAT_ and Z_HMR_ partially overlap in **a**- cells (Fig 3) in agreement with the fact that chromatin in yeast is highly flexible [38], notably between these two loci 100kb apart on the same chromosome arm. In contrast, Z_HML_ is excluded from the two other survival zones. In **a**-cells, Z_HML_ is significantly greater than in α-cells consistent with our finding that the left arm is more crumpled and flexible in a subset of nuclei (Fig 2).

In addition, we asked whether our abstract mathematical model could inform on physical properties of the chromatin fiber in yeast. First simulations (S4 Fig) suggest that the determined survival zones Z of the three mating type loci correspond to rather flexible, moderately constrained polymer fibers. For example, the survival zones of three linked loci corresponding to Z_1_ = [-1;1]×[-1;1], Z_2_ = [-1;5]×[-1;5]and Z_3_ = [3.5;6.5]×[-1.5;1.5] are closer to simulation B than to A or C (compare experimental data in Fig 3 to S4 Fig). The simulated survival zones Z2 and Z3 in our example are separated by <0.5 or less. If we assign a value of Z = 5 to the ~100kb contour length which corresponds to the separation between *MAT* (Z2) and *HMR* (Z3) the intervening fibers’s flexibility is <10kb. In future work, using different sets of multiple labels at varying distances along chromosomes, the abstract mathematical model presented in this study and polymer modeling will allow to better define chromatin fiber properties.

### Chromatin structural properties control mating type specific folding of chromosome 3

**Fig 4:**
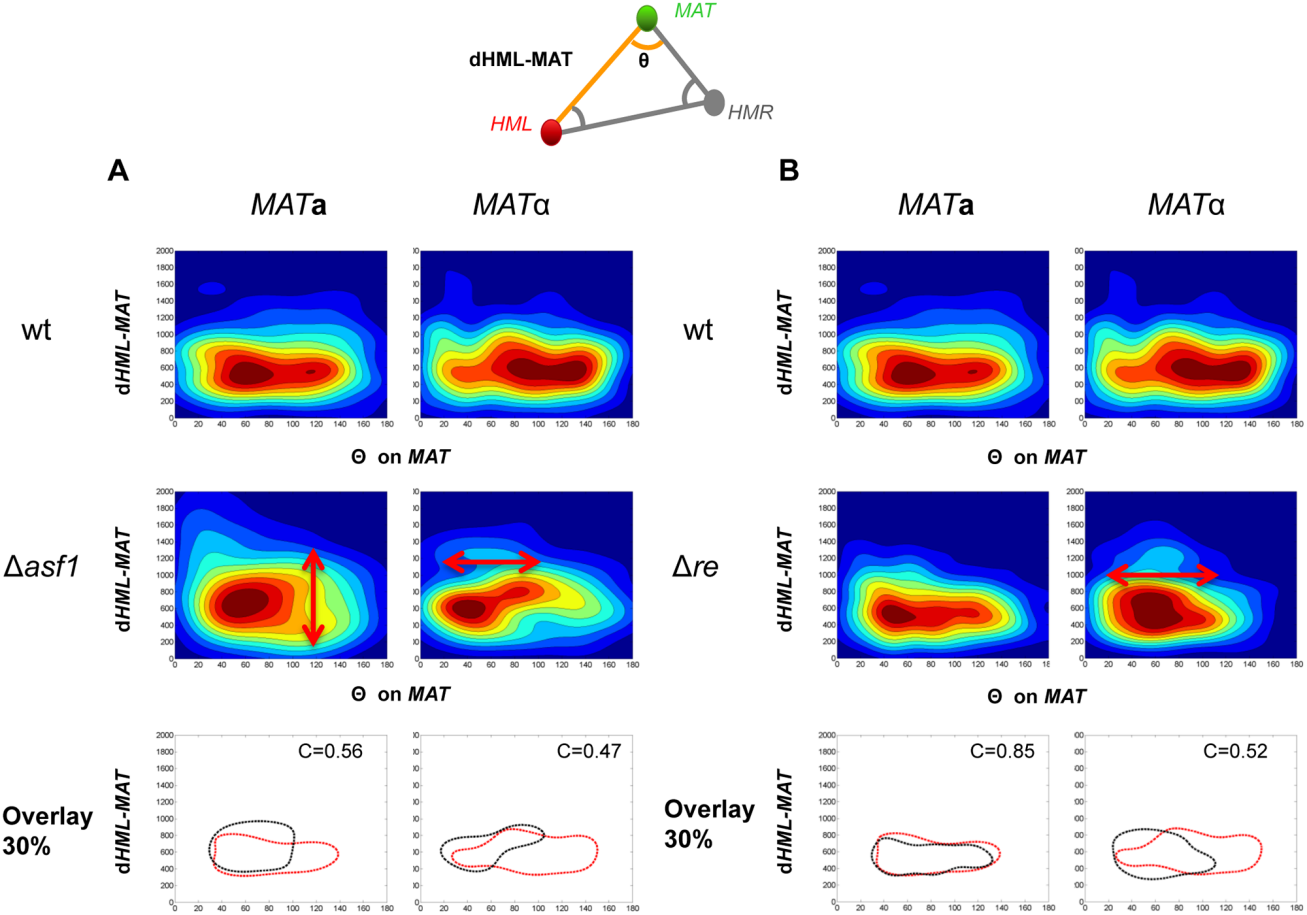
Conformation of chromosome 3 is altered in *asf1* mutant cells and in the absence of the recombination enhancer element. As in Fig 2B, *HML*, *MAT* and *HMR* were labelled. Examples of density maps (d1 = *HML-MAT*/θ at *MAT*) are shown to compare the distribution between the 3 loci in wt and mutant cells. Red arrows highlight changes. An overlay of the contours of the density area representing 30% of the analyzed *wt* (black) and mutant (red) is represented. The correlation factor c is given for 30% contour. Complete sets of density maps are shown in S5 and S7 Figs. A) wt versus *asf1*. B) wt versus a strain in which the recombination enhancer element (Δre) was deleted.

We then tested whether our abstract model could be applied to predict functional consequences in mutant cells. We deleted the *asf1* gene, coding for a histone chaperone. Asf1 was previously shown to regulate juxtaposition of the HM loci [19]. An increase in the random kinetics of the loci is expected due to general chromatin decompaction in the absence of Asf1[41]. All distances measured between the three loci on Chr3 in *asf1* strains increased by nearly 20% (S5 and S6 Figs; p<0.007). Interestingly, the distribution of angles formed around each locus was the same in wt and *asf1* mutant *MAT* **a** but varied in *MAT*α. This suggests that decompaction of chromatin in the absence of Asf1 leads to an overall extension of the chromatin fiber without compromising the folding properties of Chr3 in **a**-cells. In α-cells, however, density maps change to resemble those of wt *MAT* **a** cells (Fig 4 and S5 Fig). Computing the survival zones of *HML* relative to *MAT* and *HMR* in α-cells showed that *HML* is less constrained in *asf1* mutants than in wild type (Fig 3). Z_MAT_ and Z_HMR_ partially overlap and the zone of *HML* expands, again, similar to the situation determined in wt **a-**cells. Thus, the extended, stiffer conformation of the left arm of Chr3 in α-cells seems to be dependent on chromatin structural features brought by Asf1. The lesser impact of chromatin structure in **a**-cells suggests that the folding of the left arm of Chr3 in *MAT* **a** is intrinsic and that in α-cells certain conformations are excluded due to chromatin structural properties. To test whether a greater contact probability between *HML* and the right arm of Chr3 would favor recombination, we determined donor preference in *asf1* mutant cells. We found that in α-cells, usage of *HML* increased nearly four-fold in the absence of Asf1. Hence, reduced physical constraint of the left arm of Chr3 in a subset of cells correlates with improved recombination competence.

Finally, we asked whether the Recombination enhancer (RE), a <1kb DNA element located 17 kb to the right of *HML*α, shown to be required for recombinational competence of a large region (40 kb) near the left end of Chr3 in *MAT* **a** [22], is involved in folding of Chr3. Deletion of the RE region reduces the use of *HML* to repair *MAT* **a** from >80% to <10%[22,23]. In *MAT*α, the RE is in a heterochromatin configuration and non-functional, leaving the left arm incompetent for recombination[42]. We found that the distribution of the three loci was altered in both mating types in the absence of the RE, although the differences in density maps are more pronounced in α- than in **a-** cells (Fig 4, S6 and S7 Figs). In **a-**cells, only the most frequently observed wt conformations of the three loci remained. The relative positioning of *HML* and *MAT* varied and this was more noticeable in α-cells (S7 and S8 Figs). To verify that this effect was due to the RE element rather than the insertion of the hygromycin resistance gene we deleted the RE region using the cre/lox method. The generated density maps of the relative positions of the three loci, although in a different manner, also show differences to the wt maps. Notwithstanding that we cannot formally rule out that replacing the RE with heterologous sequences (the deletion of 803bp or the addition of 1078bp within the left arm, 29kb from the telomere) might affect chromosome folding, RE specific sequences appear to mediate some of the detected cell type dependent differences.

**Fig 5:**
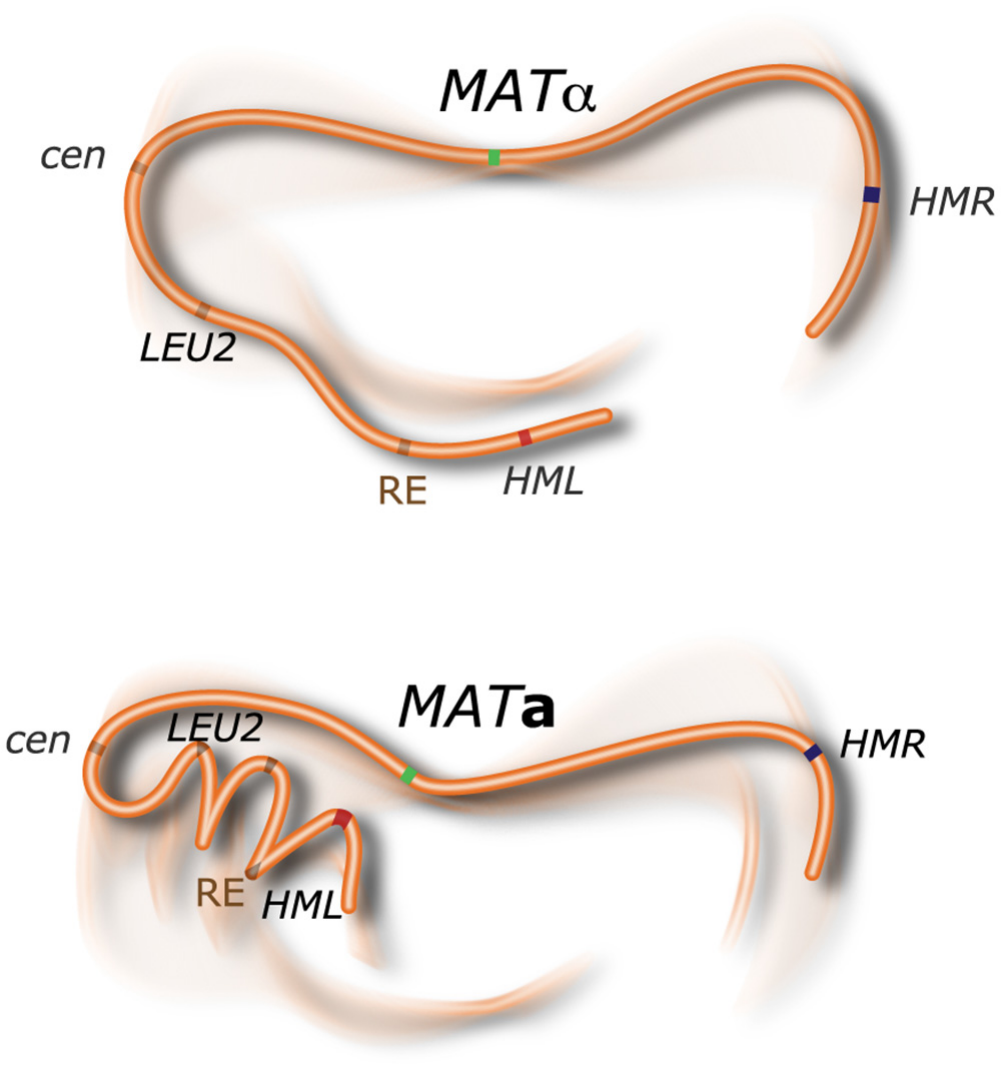
Schematic representation of the folding of chromosome 3 in *MAT*a and *MAT*α cells—only prominent features determined in this study are included. Labeled loci are indicated. Not to scale.

It was previously debated whether the RE may be involved in changing the localization or the higher-order organization of the entire left arm of Chr3 to make it more flexible in pairing with the recipient site in *MAT* **a** cells [24,25]. Our data suggest that folding of the left arm is such that at a given moment it can be in the proximity of the *MAT* locus without being pre-folded or permanently in a recombination favorable position (see model Fig 5). Thus, in *MAT* **a** cells, donor preference seems to only be imposed at commitment to recombination following cleavage of the *MAT* locus through recruitment of repair and recombination factors to RE elements or even synthesis of non-coding transcripts [23,43]. Strikingly, in α-cells, the repressed RE element appears, at least in part, to be responsible for the extended conformation of the left arm of Chr3. Hence, the heterochromatin complex at the RE may sequester part of the chromosome, an organization that could counteract looping and may limit recombination aptitude of this chromosome arm.

### Conclusion

We present a new system for labelling and visualizing a specific chromosomal site by fluorescence microscopy in living cells. We show that a small sequence element can influence the folding of an entire chromosome. Our new methodology allows three individual chromosomal sites to be imaged simultaneously in living yeast. Quantitative data can be obtained because all cells are labeled identically and permanently. The computational strategy used to evaluate the relative distribution of three objects simultaneously in 3D represents a powerful tool for studying chromosome biology and is applicable to the analysis of any three simultaneously labelled sites in any cell type. It allows identifying transient and unstable conformations of chromosomes which are statistically not the most frequently detected ones, yet may be relevant for regulating DNA processes. This view is also supported by recent studies using polymer modeling of chromatin which revealed that fluctuations in transcriptional activity correlated with probabilistic organization of the Tsix gene domain [44] and with enhancer-promoter communication via modulatory chromatin looping[45]. Our method is highly complementary to genome-wide chromosome conformation capture approaches and necessary to validate models from simulations. It is also amenable to investigation of chromosomal rearrangements governing changes in DNA-related processes in higher eukaryotes.

## Supporting Information

S1 Fig Variables and referential determination. A) To change the 3D referential, geometric variables are calculated from the microscope coordinates. A single data point is represented for each nucleus by the three components d1,d2,d3 and their associated angles. 3D data are projected onto a unique 2D plane (eg. d1/q); 9 projections can be generated from each data set. Density maps (warm colors for high density and cold colors for weak density in 10% increments) are generated for each projection in 2D. 3 Examples are shown. B) Kernel density estimation to compute de probability of the density function *f*.
(EPS)

S2 Fig Geometric organization of the mating type loci in *MAT*a and *MAT*α cells. Density maps of HM loci in *MAT* **a** (YIL30, n = 223) and *MAT*α (YIL31, n = 276) wild-type cells. A) Boxplots of pairwise distances between TetR-mRFP (*HML*), CFP-LacI (*HMR*), and YFP-λcI (*MAT*), foci (left panel) and the angles formed at each locus (right panel) in G1. Boxes represent the interquartile range (IQR) between lower and upper 25% quartiles, red bands correspond to the median distance or angle, and outliers are indicated by crosses. A median distribution Wilcoxon test was used to obtain p values. B) All generated maps are represented, and correspond to the triangulation between the position of TetR-mRFP (*HML*), CFP-LacI (*HMR*) and YFP-λcI (*MAT*) foci relative to one distance or angle at each locus. Correlation coefficients (c) obtained from the comparison between two strains at each of the ten incremental combination frequency levels is shown in Table 1. Red, dark pink and pink shades correspond to c<0.5, c<0.6 and c<0.7 respectively.
(EPS)

S3 Fig Folding of the left arm of chromosome 3 is different in *MAT*a compared to *MAT*α cells. Geometric organization of *LEU*, *HML* and *MAT* in *MAT* **a** (YIL32; n = 409) and *MAT*α (YIL33; n = 323) wild-type cells. A) boxplots of pairwise distances between TetR-mRFP (*LEU2*), CFP-LacI (*HML*), and YFP-λcI (*MAT*), foci (left panel) and the angles formed at each locus (right panel) in G1. Boxes represent the interquartile range (IQR) between lower and upper 25% quartiles, red bands correspond to the median distance or angle, and outliers are indicated by crosses. A median distribution Wilcoxon test was used to obtain p values. B) All generated density maps are represented, and correspond to the triangulation between the position of the three foci relative to one distance or angle at each locus. Correlation coefficients (c) obtained from the comparison between two strains at each of the ten incremental combination frequency levels is shown in Table 1. Red, dark pink and pink shades correspond to c<0.5, c<0.6 and c<0.7 respectively.
(EPS)

S4 Fig Simulation of the relative physical constraint of three linked loci using our abstract mathematical model to assess the properties of the chromatin fiber. Survival zones Z of three linked loci whose parameters were flexible (A), moderately constrained (B) or constrained (C) were simulated using the same iterative algorithm as for Fig 3.
(EPS)

S5 Fig Asf1 deletion increases the distance between the three mating type loci and affects the organization of the left arm of the chromosome 3 in *MAT*α and not in *MAT*a cells. Density maps represent the geometric 3D organization of the mating type loci in wild-type and *asf1* deleted *MAT* **a** and *MAT*α-cells.
(EPS)

S6 Fig Asf1 deletion increases the distance between the three mating type loci and affects the organization of the left arm of the chromosome 3 in *MAT*α and not in *MAT*a cells. A) Boxplots represent the distances measured between the three tagged loci in wild-type and *asf1 MAT* **a** (n = 242) and *MAT*α (n = 116) cells. Boxplots of pairwise distances between TetR-mRFP (*HML*), CFP-LacI (*HMR*) and YFP-λcI (*MAT*) foci (left panel) and the angles formed at each locus (right panel) in G1. Boxes represent the interquartile range (IQR) between lower and upper 25% quartiles, red bands correspond to the median distance or angle, and outliers are indicated by crosses. A median distribution Wilcoxon test was used to obtain p values. B) Correlation coefficients (c) obtained from the comparison between two strains at each of the ten incremental combination frequency levels is shown in Table 1. Red, dark pink and pink shades correspond to c<0.5, c<0.6 and c<0.7 respectively.
(EPS)

S7 Fig RE deletion alters the organization of chromosome 3 in both mating type cells differentially. Density maps represent the geometric organization of the mating type loci in wild-type and *re* deleted *MAT* **a** and *MAT*α.
(EPS)

S8 Fig RE deletion alters the organization of chromosome 3 in both mating type cells differentially. A) Boxplots representing the distances between the three tagged loci and its comparison between wild-type and *re* deleted in *MAT* **a** (n = 500) and *MAT*α (n = 324). Boxplots of pairwise distances between TetR-mRFP (*HML*), CFP-LacI (*HMR*) and YFP-λcI foci (*MAT*) (left panel) and the angles formed at each locus (right panel) in G1. Boxes represent the interquartile range (IQR) between lower and upper 25% quartiles, red bands correspond to the median distance or angle, and outliers are indicated by crosses. A median distribution Wilcoxon test was used to obtain p values. B) Correlation coefficients (c) obtained from the comparison between two strains at each of the ten incremental combination frequency levels is shown in Table 1. Red, dark pink and pink shades correspond to c<0.5, c<0.6 and c<0.7, respectively.
(EPS)

## References

[1] Duan Z, Blau CA. The genome in space and time: Does form always follow function?: How does the spatial and temporal organization of a eukaryotic genome reflect and influence its functions? BioEssays. 2012;34:800–10. 10.1002/bies.201200034 22777837 PMC3638008

[2] Rosa A, Zimmer C. Computational models of large-scale genome architecture [Internet]. 1st ed International review of cell and molecular biology. Elsevier Inc.; 2014 [cited 2014 Dec 8]. 275–349 p. Available from: http://www.ncbi.nlm.nih.gov/pubmed/24380598 10.1016/B978-0-12-800046-5.00009-6 24380598

[3] Bickmore W a, van Steensel B. Genome architecture: domain organization of interphase chromosomes. Cell [Internet]. Elsevier Inc.; 2013 3 14 [cited 2014 Jul 15];152(6):1270–84. Available from: http://www.ncbi.nlm.nih.gov/pubmed/23498936 10.1016/j.cell.2013.02.001 23498936

[4] Lieberman-Aiden E, van Berkum NL, Williams L, Imakaev M, Ragoczy T, Telling A, et al Comprehensive mapping of long-range interactions reveals folding principles of the human genome. Science. 2009;326:289–93. 10.1126/science.1181369 19815776 PMC2858594

[5] Alipour E, Marko JF. Self-organization of domain structures by DNA-loop-extruding enzymes. Nucleic Acids Res. 2012;40(22):11202–12. 10.1093/nar/gks925 23074191 PMC3526278

[6] Bohn M, Heermann DW. Repulsive forces between looping chromosomes induce entropy-driven segregation. PLoS One. 2011;6(1):1–8. 10.1371/journal.pone.0014428 PMC3014947 21245914

[7] Münkel C, Eils R, Dietzel S, Zink D, Mehring C, Wedemann G, et al Compartmentalization of interphase chromosomes observed in simulation and experiment. J Mol Biol. 1999;285:1053–65. 9887267 10.1006/jmbi.1998.2361

[8] Duan Z, Andronescu M, Schutz K, McIlwain S, Kim YJ, Lee C, et al A three-dimensional model of the yeast genome. Nature. 2010;465:363–7. 10.1038/nature08973 20436457 PMC2874121

[9] Noordermeer D, Leleu M, Schorderet P, Joye E, Chabaud F, Duboule D. Temporal dynamics and developmental memory of 3D chromatin architecture at Hox gene loci. Elife [Internet]. 2014 Jan [cited 2014 Oct 17];3:e02557. Available from: http://www.pubmedcentral.nih.gov/articlerender.fcgi?artid=4017647&tool=pmcentrez&rendertype=abstract 10.7554/eLife.02557 PMC4017647 24843030

[10] Rodley CDM, Bertels F, Jones B, O’Sullivan JM. Global identification of yeast chromosome interactions using Genome conformation capture. Fungal Genet Biol [Internet]. Elsevier Inc.; 2009;46(11):879–86. Available from: 10.1016/j.fgb.2009.07.006 10.1016/j.fgb.2009.07.006 19628047

[11] Crosetto N, Bienko M, Oudenaarden A Van. and beyond. Nat Publ Gr [Internet]. Nature Publishing Group; 2014;(12):1–10. Available from: 10.1038/nrg3832

[12] Nagano T, Lubling Y, Stevens TJ, Schoenfelder S, Yaffe E, Dean W, et al Single-cell Hi-C reveals cell-to-cell variability in chromosome structure. Nature [Internet]. Nature Publishing Group; 2013 10 3 [cited 2014 Jul 10];502(7469):59–64. Available from: http://www.pubmedcentral.nih.gov/articlerender.fcgi?artid=3869051&tool=pmcentrez&rendertype=abstract 10.1038/nature12593 24067610 PMC3869051

[13] Van den Engh G, Sachs R, Trask BJ. Estimating genomic distance from DNA sequence location in cell nuclei by a random walk model. Science. 1992;257:1410–2. 1388286 10.1126/science.1388286

[14] Dekker J. Mapping in vivo chromatin interactions in yeast suggests an extended chromatin fiber with regional variation in compaction. J Biol Chem. 2008;283:34532–40. 10.1074/jbc.M806479200 18930918 PMC2596406

[15] Bystricky K, Heun P, Gehlen L, Langowski J, Gasser SM. Long-range compaction and flexibility of interphase chromatin in budding yeast analyzed by high-resolution imaging techniques. Proc Natl Acad Sci U S A. 2004;101(47):16495–500. 15545610 10.1073/pnas.0402766101 PMC534505

[16] Kawamura R, Tanabe H, Wada T, Saitoh S, Fukushima Y, Wakui K. Visualization of the spatial positioning of the SNRPN, UBE3A, and GABRB3 genes in the normal human nucleus by three-color 3D fluorescence in situ hybridization. Chromosom Res. 2012;20:659–72. 10.1007/s10577-012-9300-5 22801776 PMC3481056

[17] Wiggins PA, Cheveralls KC, Martin JS, Lintner R, Kondev J. Strong intranucleoid interactions organize the Escherichia coli chromosome into a nucleoid filament. Proc Natl Acad Sci U S A. 2010;107:4991–5. 10.1073/pnas.0912062107 20194778 PMC2841910

[18] Bystricky K, Laroche T, van Houwe G, Blaszczyk M, Gasser SM. Chromosome looping in yeast: telomere pairing and coordinated movement reflect anchoring efficiency and territorial organization. J Cell Biol. 2005;168(3):375–87. 15684028 10.1083/jcb.200409091 PMC2171726

[19] Miele A, Bystricky K, Dekker J. Yeast silent mating type loci form heterochromatic clusters through silencer protein-dependent long-range interactions. PLoS Genet. 2009;5(5). 10.1371/journal.pgen.1000478 PMC2673037 19424429

[20] Dekker J, Rippe K, Dekker M, Kleckner N. Capturing chromosome conformation. Science. 2002;295(February):1306–11. 11847345 10.1126/science.1067799

[21] Haber JE. Mating-type genes and MAT switching in Saccharomyces cerevisiae. Genetics. 2012;191:33–64. 10.1534/genetics.111.134577 22555442 PMC3338269

[22] Wu X, Haber JE. A 700 bp cis-Acting Region Controls Mating-Type Dependent Recombination Along the Entire Left Arm of Yeast Chromosome III. 1996;87:277–85. 8861911 10.1016/s0092-8674(00)81345-8

[23] Szeto L, Fafalios MK, Zhong H, Vershon AK, Broach JR.?? 2p controls donor preference during mating type interconversion in yeast by inactivating a recombinational enhancer of chromosome III. Genes Dev. 1997;11:1899–911. 9271114 10.1101/gad.11.15.1899 PMC316413

[24] Simon P, Houston P, Broach J. Directional bias during mating type switching in Saccharomyces is independent of chromosomal architecture. 2002;21(9). 11980725 10.1093/emboj/21.9.2282 PMC125987

[25] Bressan D a, Vazquez J, Haber JE. Mating type-dependent constraints on the mobility of the left arm of yeast chromosome III. J Cell Biol [Internet]. 2004 2 2 [cited 2014 Sep 9];164(3):361–71. Available from: http://www.pubmedcentral.nih.gov/articlerender.fcgi?artid=2172233&tool=pmcentrez&rendertype=abstract 14745000 10.1083/jcb.200311063 PMC2172233

[26] Bystricky K, Van Attikum H, Montiel M-D, Dion V, Gehlen L, Gasser SM. Regulation of nuclear positioning and dynamics of the silent mating type loci by the yeast Ku70/Ku80 complex. Mol Cell Biol. 2009;29(3):835–48. 10.1128/MCB.01009-08 19047366 PMC2630671

[27] Coïc E, Richard G-F, Haber JE. Saccharomyces cerevisiae donor preference during mating-type switching is dependent on chromosome architecture and organization. Genetics [Internet]. 2006 7 [cited 2014 Oct 18];173(3):1197–206. Available from: http://www.pubmedcentral.nih.gov/articlerender.fcgi?artid=1526691&tool=pmcentrez&rendertype=abstract 16624909 10.1534/genetics.106.055392 PMC1526691

[28] Fekete R a, Chattoraj DK. A cis-acting sequence involved in chromosome segregation in Escherichia coli. Mol Microbiol [Internet]. 2005 1 [cited 2014 Oct 18];55(1):175–83. Available from: http://www.ncbi.nlm.nih.gov/pubmed/15612926 15612926 10.1111/j.1365-2958.2004.04392.x

[29] Lassadi I, Bystricky K. Tracking of single and multiple genomic loci in living yeast cells. Methods Mol Biol. 2011;745:499–522. 10.1007/978-1-61779-129-1_29 21660713

[30] Straight AF, Belmont AS, Robinett CC, Murray AW. GFP tagging of budding yeast chromosomes reveals that protein—protein interactions can mediate sister chromatid cohesion. Curr Biol [Internet]. 1996 12;6(12):1599–608. Available from: http://linkinghub.elsevier.com/retrieve/pii/S0960982202707835 8994824 10.1016/s0960-9822(02)70783-5

[31] Longtine MS, Iii AMK, Demarini DJ, Shah NG. Additional Modules for Versatile and Economical PCR-based Gene Deletion and Modification in Saccharomyces cerevisiae. 1998;961(February):953–61. 9717241 10.1002/(SICI)1097-0061(199807)14:10<953::AID-YEA293>3.0.CO;2-U

[32] Connolly B, White CI, Haber JE. Physical monitoring of mating type switching in Saccharomyces cerevisiae. Mol Cell Biol. 1988;8(6):2342–9. 2841579 10.1128/mcb.8.6.2342-2349.1988 PMC363432

[33] Parzen E. On Estimation of a Probability Density Function and Mode Emanuel Parzen. Ann Math Stat. 1962;33:1065–76.

[34] Botev ZI, Grotowski JF, Kroese DP. Kernel density estimation via diffusion. The Annals of Statistics. 2010 p. 2916–57.

[35] Robinett CC, Straight A, Li G, Willhelm C, Sudlow G, Murray A, et al In vivo localization of DNA sequences and visualization of large-scale chromatin organization using lac operator/repressor recognition. J Cell Biol. 1996;135:1685–700. 8991083 10.1083/jcb.135.6.1685 PMC2133976

[36] Roukos V, Voss TC, Schmidt CK, Lee S, Wangsa D, Misteli T. Spatial dynamics of chromosome translocations in living cells. Science [Internet]. 2013;341:660–4. Available from: http://www.ncbi.nlm.nih.gov/pubmed/23929981 10.1126/science.1237150 23929981 PMC6324928

[37] Soutoglou E, Dorn JF, Sengupta K, Jasin M, Nussenzweig A, Ried T, et al Positional stability of single double-strand breaks in mammalian cells. Nat Cell Biol [Internet]. 2007 6 [cited 2014 Oct 5];9(6):675–82. Available from: http://www.pubmedcentral.nih.gov/articlerender.fcgi?artid=2442898&tool=pmcentrez&rendertype=abstract 17486118 10.1038/ncb1591 PMC2442898

[38] Hajjoul H, Mathon J, Ranchon H, Goiffon I, Mozziconacci J, Albert B, et al High-throughput chromatin motion tracking in living yeast reveals the flexibility of the fiber throughout the genome. Genome Res. 2013;23:1829–38. 10.1101/gr.157008.113 24077391 PMC3814883

[39] Hajjoul H, Kocanova S, Lassadi I, Bystricky K, Bancaud A. Lab-on-Chip for fast 3D particle tracking in living cells. Lab Chip. 2009;9(21):3054–8. 10.1039/b909016a 19823719

[40] Nagai S, Dubrana K, Tsai-Pflugfelder M, Davidson MB, Roberts TM, Brown GW, et al Functional targeting of DNA damage to a nuclear pore-associated SUMO-dependent ubiquitin ligase. Science [Internet]. 2008 10 24 [cited 2014 Oct 1];322(5901):597–602. Available from: http://www.pubmedcentral.nih.gov/articlerender.fcgi?artid=3518492&tool=pmcentrez&rendertype=abstract 10.1126/science.1162790 18948542 PMC3518492

[41] Corpet A, Almouzni G. Making copies of chromatin: the challenge of nucleosomal organization and epigenetic information. Trends Cell Biol [Internet]. 2009 1 [cited 2014 Aug 27];19(1):29–41. Available from: http://www.ncbi.nlm.nih.gov/pubmed/19027300 10.1016/j.tcb.2008.10.002 19027300

[42] Weiss K, Simpson RT. Cell type-specific chromatin organization of the region that governs directionality of yeast mating type switching. 1997;16(14):4352–60. 9250679 10.1093/emboj/16.14.4352 PMC1170061

[43] Coïc E, Sun K, Wu C, Haber JE. Cell cycle-dependent regulation of Saccharomyces cerevisiae donor preference during mating-type switching by SBF (Swi4/Swi6) and Fkh1. Mol Cell Biol [Internet]. 2006 7 [cited 2014 Oct 18];26(14):5470–80. Available from: http://www.pubmedcentral.nih.gov/articlerender.fcgi?artid=1592702&tool=pmcentrez&rendertype=abstract 16809780 10.1128/MCB.02443-05 PMC1592702

[44] Giorgetti L, Galupa R, Nora EP, Piolot T, Lam F, Dekker J, et al Predictive polymer modeling reveals coupled fluctuations in chromosome conformation and transcription. Cell. 2014;157(4):950–63. 10.1016/j.cell.2014.03.025 24813616 PMC4427251

[45] Doyle B, Fudenberg G, Imakaev M, Mirny L a. Chromatin Loops as Allosteric Modulators of Enhancer-Promoter Interactions. PLoS Comput Biol [Internet]. 2014;10(10):e1003867 Available from: http://dx.plos.org/10.1371/journal.pcbi.1003867 10.1371/journal.pcbi.1003867 25340767 PMC4207457

